# Safety and efficacy of synchronous panniculectomy and endometrial cancer surgery in obese patients: a systematic review of the literature and meta-analysis of postoperative complications

**DOI:** 10.4274/jtgga.galenos.2019.2019.0103

**Published:** 2020-12-04

**Authors:** Anastasia Prodromidou, Christos Iavazzo, Victoria Psomiadou, Athanasios Douligeris, Nikolaos Machairas, Anna Paspala, Konstantinos Bakogiannis, George Vorgias

**Affiliations:** 1 Department of Obstetrics and Gynecology, Metaxa Memorial Cancer Hospital, Piraeus, Greece; 2 Department of Surgery, Attikon University Hospital, National and Kapodistrian University of Athens School of Medicine, Athens, Greece

**Keywords:** Panniculectomy, endometrial cancer, obesity, lymphadenectomy, wound complications

## Abstract

Panniculectomy combined with gynaecological surgery constitutes an alternative approach for endometrial cancer (EC) in obese patients. The present study aimed to assess the current knowledge concerning the safety and efficacy of combining panniculectomy in surgical management of EC. Four electronic databases were systematically searched for articles published up to May 2019. A total of five studies, of which two were non-comparative and three comparative, were included. Meta-analysis of complications among panniculectomy and conventional laparotomy group revealed no difference in either intra- or post-operative complication rates. Moreover, no difference was reported in surgical site complications (p=0.59), while wound breakdown rates were significantly elevated in the laparotomy group (p=0.02). Panniculectomy combined surgery for the management of EC appears to be a safe procedure and results in comparable outcomes compared with conventional laparotomy with regard to complications and improved wound breakdown rates.

## Introduction

Endometrial cancer (EC) remains the most common gynaecological cancer in the United States (1,2). In 2018, approximately 63,230 new cases of EC were diagnosed, with over 11,350 cancer-related deaths, while the relevant proportions from Global Cancer Statistics were 382,069 and 89,929, respectively (1,3). Moreover, obesity rates have escalated rapidly during the last decade and a continued steady increase is predicted, at least until 2030. Obese patients represent a particular patient population and thus require special management (4). Additionally, a significant correlation between obesity and the development of various malignancies including pancreatic, liver, and breast cancer and EC has been described (5).

Obesity is not only a risk factor for EC but also an important technical obstacle for its surgical management. Panniculectomy is a frequently performed procedure by plastic surgeons for the repair of abdominal wall malformations induced by massive weight loss (6). Compared to other aesthetic procedures, it has been associated with an increased risk of post-operative complications. These include wound-related complications, such as hematoma, seroma, wound infection and cellulitis or general complications such as venous thromboembolism (6). Recent studies reported a significant improvement in the incidence of complications after abdominoplasty due to improvement in operative techniques and perioperative care (7). Panniculectomy combined with gynaecological surgery has been reported as a different approach to the peritoneal cavity and has gained wide acceptance, since it provides a more favourable surgical field and the associated complications can be well managed (8).

The aim of the present review was to combine and assess the current knowledge concerning the safety and efficacy of combining panniculectomy with gynaecological surgery in the management of patients with EC and to compare the outcomes with those of conventional surgery for EC.

## Material and Methods

### Study design

The Preferred Reporting Items for Systematic Reviews and Meta-analyses (PRISMA) guidelines were followed for the design of the present systematic review and meta-analysis. The search was based on the authors’ predetermined eligibility criteria (9). An independent search of the literature was performed by three authors (C.I., A.P., V.P.) who excluded overlaps and tabulated the selected indices in a structured form. No language restrictions were assigned. Prospective and retrospective studies, which were either comparative or non-comparative and addressed outcomes of women with EC who underwent surgical staging with concomitant panniculectomy were considered eligible for inclusion in the present systematic review. Reviews, case reports, abstracts and animal studies were excluded from analysis and tabulation.

### Search strategy and data collection

**Figure 1:**
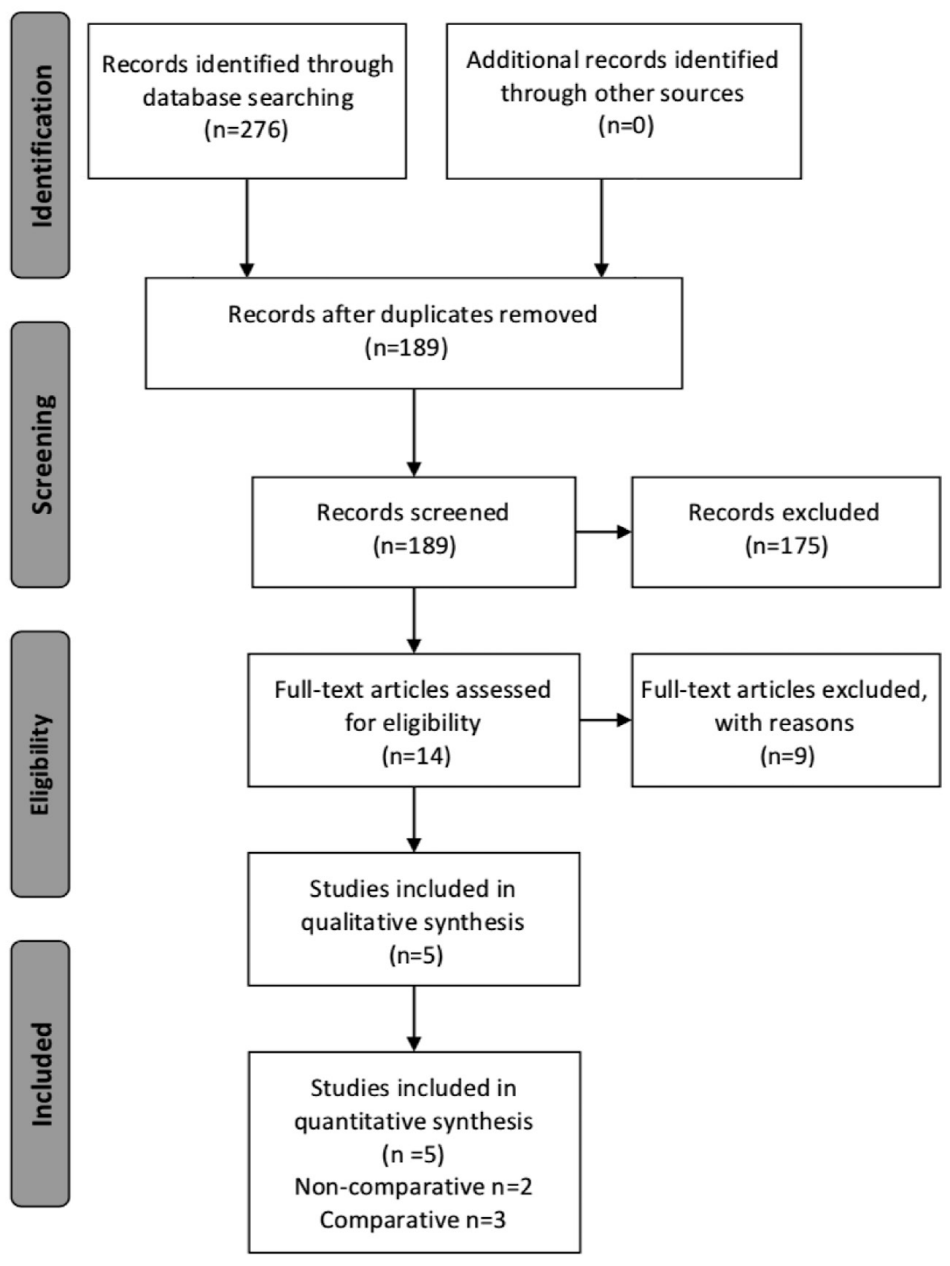
Search flow diagram

A systematic search of the literature was conducted for articles published up to May 2019. Databases searched were PubMed (1966-2019), Google Scholar (2004-2019), Scopus (2004-2019), and the ClinicalTrails.gov database, along with the references of the articles retrieved in full text. The key words which were used for the search were: “EC”, “uterine cancer”, “corpus cancer”, “panniculectomy”, “apronectomy”, “lymphadenectomy”. A limited number of keywords were used with the intent to assess an eligible number, which could be easily searched and, at the same time, minimizing the potential loss of eligible articles. Articles that fulfilled or were considered to fulfil the eligibility criteria were retrieved in full text. All studies with more than 10 cases of obese women with EC, aged >18 years, who underwent a combination of surgical management for EC with panniculectomy, were included. Comparative and non-comparative studies reporting at least one postoperative outcome including operative time (OT), estimated blood loss (EBL), length of hospital stay (LOS), resected lymph nodes count (pelvic or para-aortic) and incidence of complications, were considered eligible for inclusion. Comparative studies which presented outcomes of obese patients who had surgery for EC with additional panniclulectomy versus those who had did not undergo panniculectomy and received only conventional EC-related surgical procedures were also considered eligible for inclusion. The meta-analysis was based on the assessment of the complication rates as the primary outcome. The stages of selection of the recruited articles are schematically presented in Figure 1 which depicts the PRISMA flow diagram.

### Quality assessment

The Methodological Index for Non-Randomized Studies (MINORS) was utilized to assess the quality of the recruited studies (10). MINORS consists of a quality assessment tool which was designed to estimate non-randomized studies methodological adequacy. Due to the fact that all the studies included in the present meta-analysis were non-randomized, the MINORS scale was used.

### Statistical analysis

The RevMan 5.3 software (Copenhagen: The Nordic Cochrane Centre, The Cochrane Collaboration, 2011) was used for statistical meta-analysis. Confidence intervals (CI) were set at 95%, whereas mean difference and odds ratios (OR) were used for the analysis. In all the examined parameters, the DerSimonian-Laird random effect model was utilized, due to the expected significant heterogeneity of the studies (11). P-value <0.05 was set as the cut-off for statistical significance. Due to the fact that heterogeneity of the included studies may influence the methodological integrity of the tests, publication bias was not tested.

## Results

Due to the high heterogeneity of the included studies and more specifically the discrepancy with regards to the way of interpretation of the examined parameters in comparative studies, meta-analysis of the results was precluded for most of the parameters. A meta-analysis was specifically performed for overall and surgical site complications. Therefore, for the remaining parameters a meticulous systematic review was conducted. The analysed indices were tabulated in three structured tables as follows: Table 1, included the main characteristics of comparative and non-comparative studies; Table 2, 3 recorded the main characteristics of the patients and the main intra- and post-operative outcomes, respectively.

### Excluded studies

A total of nine studies were excluded from this systematic review. More specifically, six reported outcomes with regards to gynecologic oncology surgical procedures combined with panniculectomy were initially considered eligible. After retrieving the full text, it was noticed that no separated outcomes for patients operated for EC were provided and the studies was excluded (12,13,14,15,16,17). Additionally, Cosin et al. (18) and Micha et al. (19) were not included, due to limited patient numbers. Finally, in the study by Patibandla et al. (20) insufficient data made it ineligible for inclusion.

### Included studies

Five studies, which reported patients who underwent surgery for EC with or without panniculectomy were finally included in the present study (21,22,23,24,25). Specifically, two studies were non-comparative and included 33 patients (21,22) while the remaining three studies were comparative studies and evaluated results of 65 patients who received simultaneous laparotomy for EC and panniculectomy (Panniculectomy group) versus 416 who underwent laparotomy only for EC (Laparotomy group) (23,24,25).

### Quality assessment

The MINORS quality assessment revealed methodological adequacy of the included studies and the presence of low heterogeneity with regards to their quality. A mean score of 13.8 (standard deviation: 4.5) with a respective median score of 16 (range: 8-18) (Table 1) were calculated.

### Intraoperative and postoperative outcomes

A total of 98 patients underwent surgery for EC and simultaneous panniculectomy. Seventy-seven women had stage I/II EC and nine had stage III/IV EC, according to FIGO classification, while for the remaining 12 patients staging was not reported (Table 2). Data of perioperative outcomes with regards to patients who underwent combined surgery, showed a median (range) OT of 247.7 (90-355) minutes and a median (range) EBL of 486.5 (50-1200) mL. The incidence of intraoperative complications was 8.5% (n=5/59). Median (range) LOS was 6 (3,4,5,6,7,8,9,10,11,12,13,14,15) days. Concerning postoperative complications, a total of 25 patients (25.5%) presented with non-surgical site complications, whereas 26 patients (26.5%) had surgical site complications. Among them, 13 were wound infections, six had cellulitis, and three wound breakdowns were reported while for the remainder data concerning the type of complication was not available (Table 3).

With regards to the comparative studies, as shown in Table 3, no difference in mean body mass index (BMI) among patients who underwent combined surgery and those who underwent only laparotomy was reported by the study of Wright et al. (24) whereas Ramzan et al. (23) and Eisenhauer et al. (25) reported significantly higher BMI in the panniculectomy group. Intraoperative outcomes revealed a significantly prolonged OT in the panniculectomy group in comparison to laparotomy group in all of the included studies (p<0.001) whereas EBL was not significantly different (p>0.05). No difference was reported with regards to LOS (p>0.05). Data from two of the studies showed that pelvic lymph node dissection was performed in 85.2% of patients in the panniculectomy group and in 57.2% in the laparotomy group (24,25). Eisenhauer et al. (25) reported a significantly elevated count of harvested pelvic lymph nodes in patients in the panniculectomy group (p=0.001). In contrast, Wright et al. (24) did not find a difference in mean pelvic lymph node count between the two groups (p=0.199). A total of 61% of patients from the panniculectomy group and 44% from the laparotomy group had para-aortic lymphadenectomy (24,25). Wright et al. (24) noted a significantly higher proportion of para-aortic lymph nodes dissected in the panniculectomy group when compared to women who underwent simple laparotomy (p=0.032). On the contrary, median para-aortic lymph node count did not differ among the two group of patients as reported by Eisenhauer et al. (25) (p=0.18).

**Figure 2:**
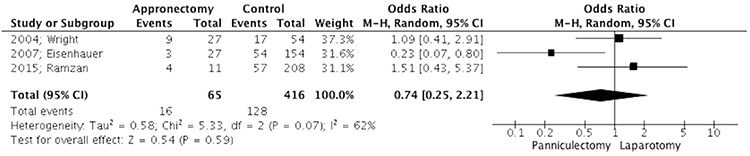
Forest plot depicting surgical site complication CI: Confidence interval

**Figure 3:**
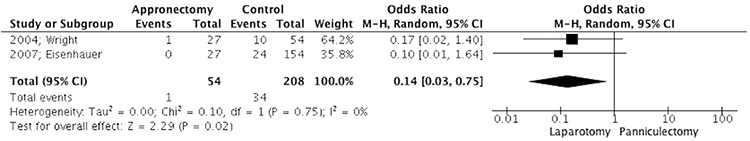
Forest plot depicting wound breakdown rates CI: Confidence interval

Meta-analysis of complications revealed no difference in overall complication rates, when surgical site complications were excluded, among the two groups either in intra-operative or post-operative complications (481 cases, OR: 1.06 95% CI: 0.31-3.58 p=0.93 and 300 cases OR: 1.49 95% CI: 0.46-4.82 p=0.51, respectively). Concerning surgical site complications, the overall effect did not reveal a significant difference between the Panniculectomy and Laparotomy groups (481 cases OR: 0.74 95% CI: 0.25-2.21 p=0.59) (Figure 2). When incision related parameters, such as wound infection, cellulitis and wound breakdown were separately analyzed, statistical significance was noted only in wound breakdown rates, which were found to be significantly elevated in patients who did not undergo panniculectomy (262 cases OR: 0.14 95% CI: 0.03-0.75 p=0.02) (Figure 3). The incidence of wound infection and cellulitis did not differ between the two groups (262 cases OR: 0.53 95% CI: 0.11-2.44 p=0.41 and 262 cases OR: 0.93 95% CI: 0.05-16.20 p=0.96, respectively).

## Discussion

The main aim of this systematic review was to evaluate the efficacy and safety of panniculectomy in selected cases who underwent surgery for EC by assessing the main peri-operative outcomes reported by the recruited studies. In patients undergoing combined surgery, the median OT in the laparotomy without panniculectomy group was 206.7 minimum and median EBL was 486.5 mL, while there was a similar prevalence of approximately 26% observed in non-surgical site and surgical site complications among the included patients. Despite the prolonged OT in the panniculectomy group, EBL and LOS were comparable among patients who had panniculectomy combined surgery and conventional EC surgery. Additionally, meta-analysis revealed no difference in either non-surgical site or in surgical site complications, whereas subgroup analysis of wound infection, cellulitis and wound breakdown revealed a difference only in the incidence of the latter.

Obese patients who undergo surgery for EC are potentially at higher risk of intra- or post-operative complications due to excess subcutaneous fat. To that end, application of panniculectomy has gained popularity as an additional procedure during surgery for the treatment of gynaecological malignancies, and more specifically, EC. Panniculectomy is a particular type of abdominoplasty, and tends to be less radical than other methods of abdominoplasty. It was initially applied in multiparous women who presented with a prominent apron in their abdominal wall (26). Favourable cosmetic and medical outcomes have also been reported in obese patients or patients that lost weight and suffer from an excess abdominal skin (26,27). The procedure involves removal of as much excess adipose tissue as can be resected without leaving tension of the remaining tissue at closure. The rectus muscle and its sheath, which is usually morbid in patients with large pannus, is then reconstructed (28). Umbilicus preservation is attempted. Specifically, a scalpel is usually used for transverse skin incisions and an electrosurgical source is used for the excision of the underlying subcutaneous tissue. The procedure is performed before entering the peritoneal cavity, entry to which is made through a midline incision. At the end of the procedure, the abdominal flaps are closed with sutures to the subcutaneous tissue, drainage is placed and the skin is also sutured.

In the present study, about one fourth of patients who underwent gynecological surgery combined with panniculectomy presented with either non-surgical site or surgical site post-operative complications. Despite the fact that this rate could be considered relatively high, there are in agreement to those reported by other studies, which examined the efficacy of panniculectomy combined surgery in obese patients with gynecological malignancies (29,30). More specifically, a retrospective study by Rasmussen et al. (29) evaluated post-operative complications after panniculectomy combined with a gynecologic procedure, such as hysterectomy (simple or radical) or laparotomy for ovarian cancer staging. The overall complication rates were 31.3% and most were superficial cellulitis (28.3%) (29). Furthermore, according to a comparative study by Forte et al. (30) no significant differences were detected with regards to overall and wound-related complications among patients who underwent panniculectomy combined hysterectomy and those who had hysterectomy alone. Similarly, the present meta-analysis revealed no difference in wound infection rates and cellulitis among the patients included. On the contrary, the authors suggested that the significantly increased wound breakdown rates in the simple laparotomy group are possibly due to the excessive pannus that remained after surgery in this group.

Lymph node yield could be considered as a quantitative method of evaluation of the efficacy of panniculectomy, which results in an improvement in the vision of the surgical field during surgical management of EC. In that setting, a potential increase in the count of resected lymph nodes could indicate the superiority of panniculectomy combined surgery for EC (20). Eisenhauer et al. (25) reported the resected lymph node count was significantly increased in patients who also had panniculectomy compared to those who underwent simple laparotomy. However, no difference was detected among the two groups by Wright et al. (24). However, data is still limited and further studies are warranted to resolve this question.

Considering that panniculectomy is a relatively rare procedure for non-cosmetic indications, combined with simultaneous advances in minimally invasive procedures in the management of EC, there may be increasing confusion concerning the exact indications for this procedure. Nonetheless, in case of obese and extremely obese patients, minimally invasive surgery still remains challenging, because of the technical difficulties that are related to excess fat and the impact on the visualization, the radicallity of the procedure and the OTs (31). Panniculectomy combined procedures could be considered as an alternative for patients with high BMI. Outcomes from the included studies imply a good safety profile for the procedure, despite the fact that they derive from small retrospective studies. Additionally, the precise indications of the procedure, the BMI above which patients could benefit from the procedure, along with the extent of pannus are not properly identified. To that end, Ramzan et al. (23) suggested that patients with BMI of more than 60 kg/m^2^ as well as patients who will require lymph node dissection could be considered as candidates for panniculectomy. However, further randomized controlled trials, which evaluate the outcomes after minimally invasive surgery, simple laparotomy and panniculectomy-combined laparotomy are needed, in order to identify the most appropriate approach according to each BMI and designate the candidates for panniculectomy.

### Study Limitation

There are some limitations that need to be addressed. First of all, the retrospective nature of the articles included, along with their heterogeneity, constitute significant limitations. Furthermore, all the studies included were non-randomized which further limits the interpretation of the exact role of patients’ characteristics as confounders. Concerning the comparative studies, the control groups were not matched with regards to patient characteristics. Consequently, the panniculectomy group included patients with significantly greater BMI compared to control in two of the recruited studies, is an additional limitation of our findings. Furthermore, the definition of obesity was not consistent between the included studies. Therefore, potential bias with regards to selection and attrition bias and selective reporting may skew our outcomes. In some studies, report of the outcomes measures was inadequate, especially with regards to continuous parameters such as lymph node yield, in which outcome reports different methods were utilized for the interpretation of the results and thus some were not included in the analysis. Accordingly, oncological outcomes were underreported by the included studies. More specifically, disease free survival and overall survival rates were only available in the study by Wright et al. (24) who reported comparable rates among the two groups. Furthermore, the small sample sizes of the included patients in each group constituted a further limitation of our study. Finally, assessment of publication bias was not feasible concerning the small size of the studies included.

## Conclusion

Panniculectomy combined surgery for the management of EC can be considered a safe procedure in selected patients and presents with comparable outcomes to conventional laparotomy procedures with regard to non-surgical and surgical site complications and improved wound breakdown rates. However, those outcomes must be cautiously interpreted because of the limited number of studies included in this meta-analysis and their retrospective nature. To the best of our knowledge, the present study is the only one in this field which assessed post-operative results in patients who had panniculectomy combined surgery for EC in obese patients. There is a need for further, larger-volume studies with the intention of defining the optimal approach, specifying the group of obese patients with EC who could benefit from panniculectomy and elucidate the efficacy of panniculectomy in enhancing the lymph node yield in those patients.

## Figures and Tables

**Table 1:**
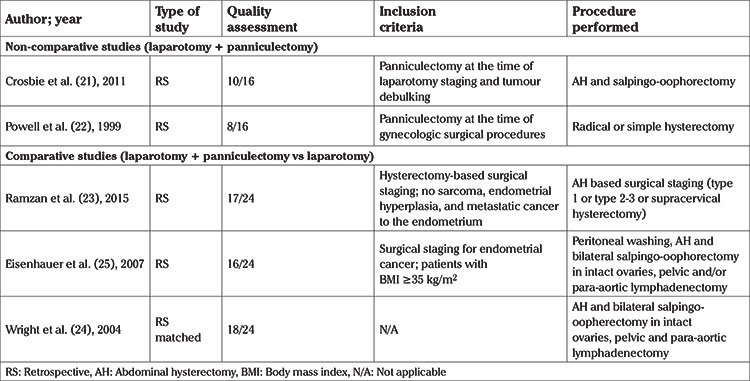
Characteristics of included studies

**Table 2:**
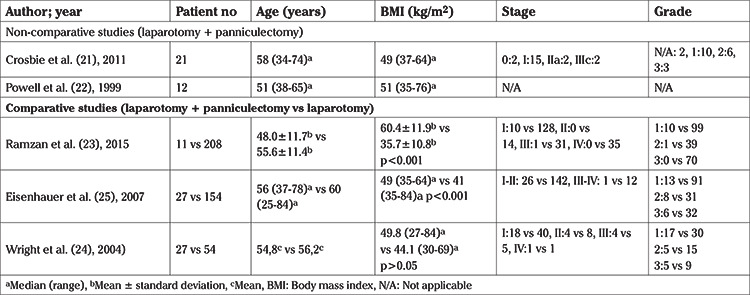
Characteristics of included patients

**Table 3:**
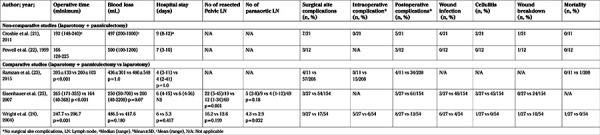
Main intra-and postoperative outcomes
